# Avian Influenza H5N6 Viruses Exhibit Differing Pathogenicities and Transmissibilities in Mammals

**DOI:** 10.1038/s41598-017-16139-1

**Published:** 2017-11-24

**Authors:** Zongzheng Zhao, Zhendong Guo, Chunmao Zhang, Lina Liu, Ligong Chen, Cheng Zhang, Zhongyi Wang, Yingying Fu, Jiaming Li, Huabin Shao, Qingping Luo, Jun Qian, Linna Liu

**Affiliations:** 1 Institute of Military Veterinary, Academy of Military Medical Sciences, 666 West Liuying Road, Changchun, 130122 Jilin, China; 2 College of Veterinary Medicine, Hebei Agricultural University, 2596 lucky south street, Baoding, 071000 Hebei, China; 3 Key Laboratory of Prevention and Control Agents for Animal Bacteriosis, Institute of Animal Husbandry and Veterinary, Hubei Academy of Agricultural Sciences, Wuhan, China

## Abstract

Since 2013, highly pathogenic avian influenza H5N6 viruses have emerged in poultry and caused sporadic infections in humans, increasing global concerns regarding their potential as human pandemic threats. Here, we characterized the receptor-binding specificities, pathogenicities and transmissibilities of three H5N6 viruses isolated from poultry in China. The surface genes hemagglutinin (HA) and neuraminidase (NA) were closely related to the human-originating strain A/Changsha/1/2014 (H5N6). Phylogenetic analyses showed that the HA genes were clustered in the 2.3.4.4 clade, and the NA genes were derived from H6N6 viruses. These H5N6 viruses bound both α-2,3-linked and α-2,6-linked sialic acid receptors, but they exhibited different pathogenicities in mice. In addition, one virus was fully infective and transmissible by direct contact in guinea pigs. These results highlight the importance of monitoring the continual adaptation of H5N6 viruses in poultry due to their potential threat to human health.

## Introduction

Since the first human H5N6 virus infection was reported in China^1^, H5N6 viruses have caused sporadic infections in humans; at least 17 human infections have been reported in China. The emergence of human H5N6 influenza virus infection has raised global concerns regarding potential threats to human health.

The first H5N6 virus was detected in mallards in North America in 1975^2^. In the past, H5N6 viruses exhibited low pathogenicity and had little impact on the poultry industry and human health, including in Germany in 1984^3^ and in Sweden in 2002^4^. However, the recently emerged Asian H5N6 strain showed high pathogenicity, and a series of poultry outbreaks resulting from H5N6 recently occurred in China, Laos, Vietnam, Korea, and Japan^5–10^.

H5N6 initially arose from the reassortment of H5N1 and H6N6. The second reassortment may have occurred between H5N6 and ZJ-HJ/07-like H9N2 viruses^11^, which are similar to H7N9^12^ and H10N8 viruses^13^. In 2015, the third reassortment was generated by a deletion in the NA protein at residues 59–69, resulting in novel H5N6 viruses that were more likely to cross species barriers to infect humans^14^. Currently, H5N6 has replaced H5N1 as the dominant avian influenza virus subtype in poultry in southern China. Furthermore, H5N6 was also detected in migratory waterfowl prior to the first human case^15,16^. Migratory waterfowl, which transverse long distances, played a major role in the geographical spread of H5N6. Chickens and ducks functioned as “vessels” to deliver H5N6 from avian species to humans^14,17^, and H5N6 continued to evolve to adapt to mammals. We recently isolated three H5N6 viruses from domestic ducks and chickens in Hubei province, China. However, the zoonotic capabilities and pathogenicities of H5N6 viruses in poultry remained unknown. In the present study, we used phylogenetic analysis and evaluated the receptor-binding properties, pathogenicities and transmissibilities of H5N6 viruses. These studies expand our understanding of the pathogenicity and transmissibility of H5N6 viruses and will aid in influenza pandemic preparedness efforts.

## Results

### Phylogenetic analysis of surface genes

Nucleotide sequences of the haemagglutinin (HA) and neuraminidase (NA) genes (GenBank accession numbers MG029168 to MG029173) in three H5N6 viruses, A/duck/Hubei/XY01/2016 (abbreviated as DK01), A/chicken/Hubei/XY165/2016 (abbreviated as CK165) and A/chicken/Hubei/XY918/2016 (abbreviated as CK918), were compared with those of known influenza viruses in the GenBank database.

**Figure 1:**
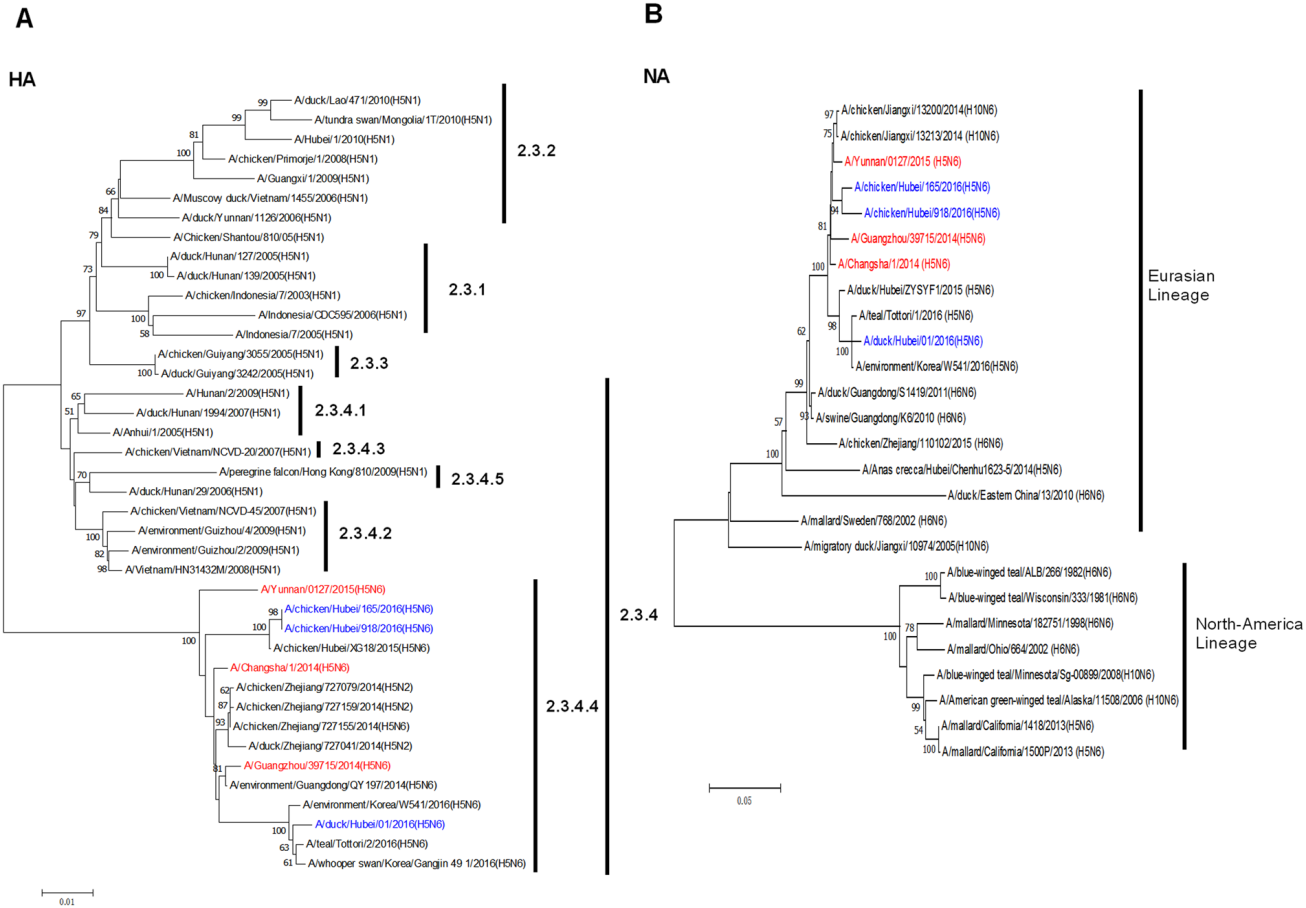
Phylogenetic analysis of HA and NA genes. Phylogenetic trees of the HA (**A**) and NA (**B**) genes were constructed using the distance-based neighbor joining method in MEGA7.0.21 software. The reliability of the trees was assessed by bootstrap analysis. Horizontal distances are proportional to genetic distances. The H5N6 viruses analyzed in the present study are indicated in blue. Human H5N6 viruses are indicated in red.

Phylogenetic analyses demonstrated that the HA genes of the three H5N6 viruses were clustered in the 2.3.4.4 clade (Fig. 1A). Sequence analysis elucidated nineteen amino acid differences in the DK01, CK165 and CK918 HA genes (G5S, K145R, E80K, A88T, E102D, P120L, T131E, S132T, L142Q, K144V, R173G, E189A, V213L, S227E, D240N, N277H, 328K delete, N405S, G528S). The NA genes of the three H5N6 viruses were closely related to those of the H6N6 and H10N6 viruses A/duck/Guangdong/S1419/2011 (H6N6) and A/chicken/Jiangxi/13213/2014 (H10N6) and were derived from N6-like Eurasian virus lineages (Fig. 1B). In total, 11 amino acid deletions were detected in the NA stalk regions (positions 59 to 69) of the three H5N6 viruses. In addition, the HA and NA genes of the three H5N6 viruses were also closely related to those of the human isolates A/Changsha/1/2014 (H5N6), A/Guangzhou/39715/2014 (H5N6) and A/Yunnan/0127/2015 (H5N6), as they shared 96–98% nucleotide identity. These results indicate that the three H5N6 viruses share original ancestors similar to those of human-originating H5N6 viruses.

### H5N6 viruses exhibited different binding affinities for avian and human sialic acid receptors

**Figure 2:**
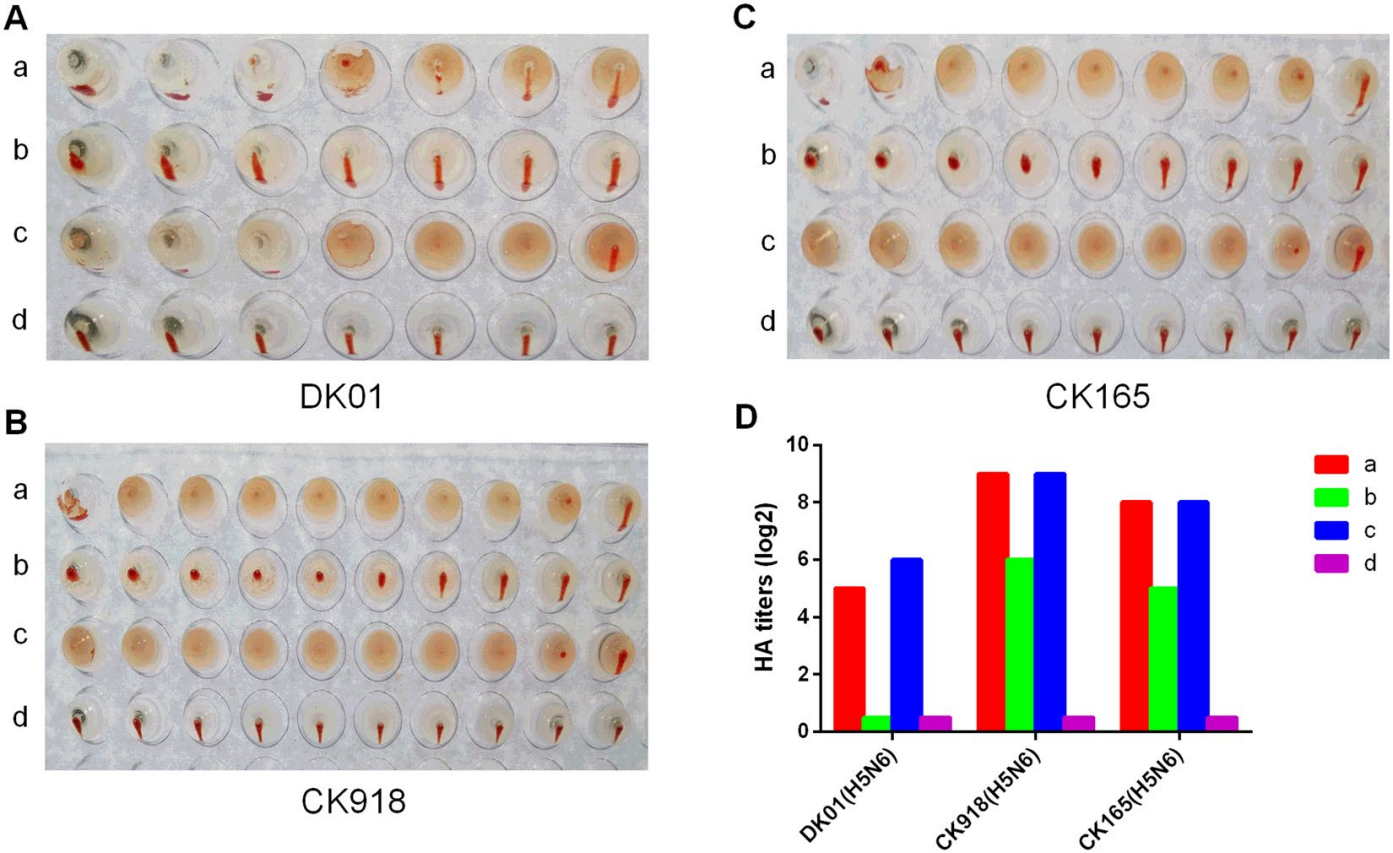
Agglutination activities of the H5N6 viruses for various red blood cells. Four types of red blood cells: a Chicken red blood cells (with α-2,3-linked sialic acid receptors and α-2,6-linked sialic acid receptors). b, Chicken red blood cells treated with α-2,3-sialidase (with only α-2,6-linked sialic acid receptors). c, Sheep red blood cells (with only α-2,3-linked sialic acid receptors). d, Chicken red blood cells treated with VCNA (no receptors). Figure 2A,B and C show the agglutination activities of DK01, CK918 and CK165 for the four types of red blood cells, respectively. Figure 2D represents the HA titers of DK01, CK918 and CK165 in the four types of red blood cells.

We evaluated the receptor-binding specificities of the three H5N6 viruses (DK01, CK918 and CK165) using an HA assay. The surface of chicken red blood cells (cRBCs) contains α-2,3-linked and α-2,6-linked sialic acid receptors. cRBCs treated with α-2,3-sialidase contain only α-2,6-linked sialic acid receptors, cRBCs treated with vibrio cholera neuraminidase (VCNA) contain no receptors, while the surface of sheep red blood cells (sRBCs) contain only α-2,3-linked sialic acid receptors. As expected, all the H5N6 viruses could agglutinate untreated cRBCs and sRBCs but not VCNA-treated cRBCs (Fig. 2A,B and C, row a, row c, row d). CK918 and CK165 showed some binding affinity for α 2,3-sialidase-treated cRBCs (Fig. 2B and C, row b), while DK01 showed very weak hemagglutination of α 2,3-sialidase-treated cRBCs (Fig. 2A, row b). The HA titers are displayed in Fig. 2D. These results suggested that all the H5N6 viruses maintained affinity for avian-like (α-2,3) receptors. Moreover, CK918 and CK165 exhibited affinity for human-like (α-2,6) receptors, suggesting their potential to infect humans.

### Pathogenicities of the H5N6 viruses in mice

**Figure 3:**
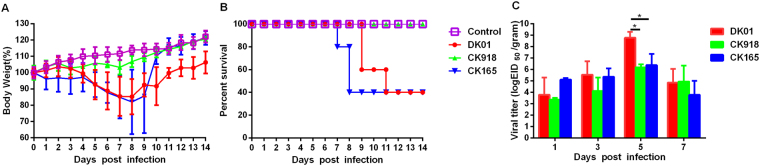
Pathogenicities of the H5N6 viruses in mice. Five mice per group were intranasally inoculated with the H5N6 viruses at 10^6^ EID_50_. (**A**) Their body weights were monitored daily for 14 days. The values represent the average scores of overall body weight loss compared with the initial body weight ± standard deviations (SD). (**B**) The percentage of survival values were calculated by observing the infected mice. (**C**) Lungs were collected from the infected mice (n = 3) on the indicated days post-infection (dpi), and viral titers were determined in 9-day-old specific pathogen-free embryonated eggs.

We evaluated the pathogenicities of the three H5N6 viruses in a mouse model. Mice inoculated with DK01 and CK165 rapidly lost approximately 20–25% of their weight, whereas CK918-inoculated mice experienced no substantial body weight loss (Fig. 3A). Mice infected with DK01 or CK165 exhibited 60% mortality, while the CK918 infection was not lethal (Fig. 3B).

We next investigated viral titers in the lungs of mice infected with the viruses at a dose of 10^6^ EID_50_. All three of the H5N6 viruses could be detected, with titers ranging from 10^3.4^ to 10^8.8^ EID_50_/gram (Fig. 3C). The viral titers gradually increased and reached higher levels at 5 dpi. CK165 and CK918 were detected at similar viral titers in the lungs of the infected mice. However, the DK01 viral titer was approximately 10^8.8^ EID_50_/gram at 5 dpi, which was 300-fold higher than that of CK165 and CK918 (p < 0.01 and p < 0.001, respectively, n = 3). The viral titers in the lungs of all the infected mice decreased at 7 dpi.

**Figure 4:**
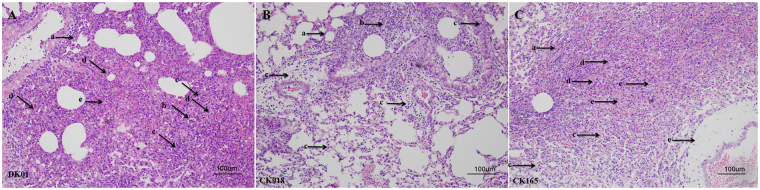
Histopathology analysis. Lungs of the infected mice were fixed with formalin, embedded in paraffin and stained with hematoxylin and eosin. (Arrow a): alveolar wall thickening; (arrow b): epithelial cell shedding; (arrow c) lymphocyte infiltration; (arrow d) alveolar cavity decreasing or even disappearing; (arrow e) epithelial cell swelling and rarefaction of cytoplasmic structures.

Additionally, we also performed histopathological analysis, and the lungs of all the H5N6-infected mice showed alveolar wall thickening, epithelial cell shedding and lymphocyte infiltration (Fig. 4A,B and C). However, the alveolar sacs appeared to have either collapsed, filled with cellular infiltrates or a combination of both in CK165- and DK01-infected mice (Fig. 4A and C). Histological analysis demonstrated that the mice infected with DK01 and CK165 exhibited more severe histopathological changes than CK918-infected mice.

In summary, based on our mouse infection studies, DK01 and CK165 were more virulent than CK918.

### Varying transmissibilities of the H5N6 viruses in guinea pigs

**Figure 5:**
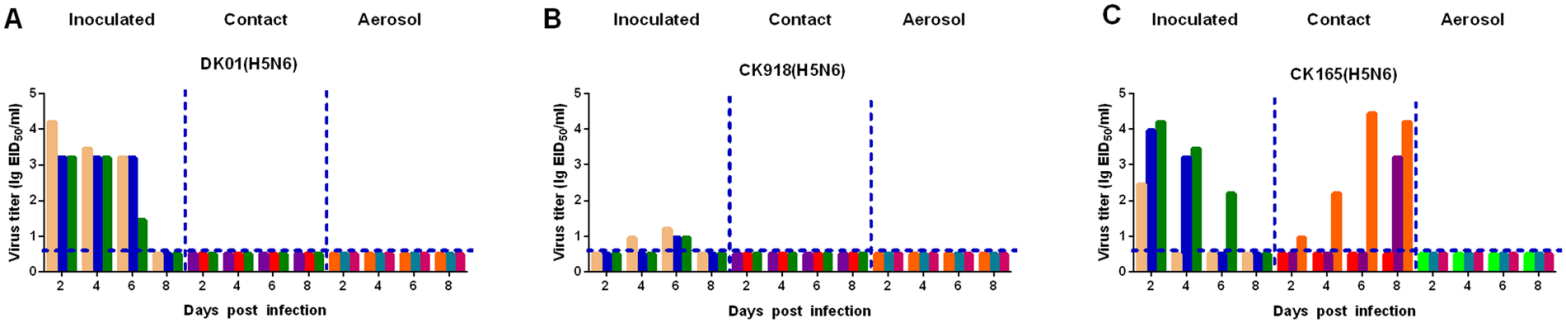
Horizontal transmission of the H5N6 viruses between guinea pigs. Groups of three guinea pigs were inoculated with the indicated viruses. The next day, the inoculated animals were paired by co-housing with direct-contact guinea pigs; aerosol-transmission animals were also housed in a wire-frame cage adjacent to the infected guinea pigs. Nasal washes were collected from all the animals for virus shedding detection every other day beginning on day 2 after the initial infection. Each color bar represents the virus titer in an individual animal. Dashed lines indicate the lower limit of virus detection.

Guinea pigs were used to investigate the transmissibility of the three H5N6 viruses. Nasal washes from all the inoculated, direct-contact and aerosol-transmission groups were collected and titrated for virus in eggs. DK01 and CK918 viruses were detected in only the nasal washes from all the inoculated guinea pigs, indicating that no virus transmission occurred (Fig. 5A and B). CK165 was transmitted to two guinea pigs via a direct contact route but not via aerosol (three animals were examined in each group) (Fig. 5C). These findings demonstrated that CK165 had acquired the ability to spread via contact transmission but not via aerosol in guinea pigs.

## Discussion

In this study, we found that the three H5N6 viruses exhibited different pathogenicities and transmissibilities in mammals. Each of the three H5N6 viruses acquired varying degrees of binding affinity for human-like receptors, and CK165 could transmit between guinea pigs by direct contact but not via aerosol.

Influenza A viruses can infect a variety of animal species; the infection begins with the HA glycoprotein binding sialic acid receptors on the surface of host cells^18,19^. The receptor-binding specificities of influenza viruses play a major role in interspecies transmission. Avian influenza viruses, including HPAI H5 and H7 viruses, have occasionally broken the species barrier and infected humans, but they have not been able to disseminate. The major reason underlying their limited human transmissibility is the weak affinity of these viruses for α-2,6-linked sialic acid receptors^18^. For efficient person-to-person transmission, viruses must adapt human receptor-binding abilities.

All three of the H5N6 viruses exhibited loss of glycosylation at residue 158 in HA, which was shown to be responsible for H5N1 binding to human-type receptors (α-2,6) in previous studies^18^. In this study, we found that the three H5N6 viruses had acquired human-type (α-2,6) receptor-binding affinity. Therefore, the loss of glycosylation at residue 158 in HA may contribute to α-2,6 receptor binding. CK918 and CK165 showed higher α-2,6 receptor-binding abilities than DK01, and they may have acquired some significant amino acid mutations in HA to confer human-type receptor-binding. Four amino acid differences in the HA genes of DK01, CK165 and CK918 were detected within the receptor-binding site (RBS) of HA^20,21^: two in the 130 loop (T131E and S132T, H3 numbering), one in the 190 helix (E189A), and one in the 220 loop (S227E). Thus, these mutations may contribute to human-type receptor-binding affinity.

In our mouse study, the pathogenicities of the three H5N6 viruses were different. DK01 exhibited a higher growth property and pathogenicity than CK918; CK165, which had growth comparable to that of CK918, also exhibited a higher pathogenicity than CK918. Thus, while increased growth properties of influenza viruses may contribute to their pathogenicity in mice, that may not be the sole underlying pathogenetic mechanism.

Previous studies showed that clade 2.3.4.6 HPAI H5N2 viruses could transmit efficiently in guinea pigs, but clade 2.3.4.4 HPAI H5N8 viruses lacked transmissibility^22^. Our data together with previous studies clearly showed that clade 2.3.4.4 HPAI H5N6 viruses were transmissible in guinea pigs by direct contact^23^, which indicated that HPAI H5N6 viruses pose a potential pandemic threat.

In summary, three avian influenza H5N6 viruses exhibited varying degrees of affinity for human-type (α-2,6) receptors, replicated well in a mouse model, and one was capable of transmitting in guinea pigs by direct contact. Notably, H5N6 viruses have maintained their adaptation for mammals, and human isolates have been found to reassort with H9N2 viruses. Therefore, H5N6 viruses may become the next potential candidates for global dissemination.

## Materials and Methods

### Ethics statement

All animal studies were conducted in strict accordance with the guidelines of animal welfare of the World Organization for Animal Health. Experimental protocols involving animals were approved by the Animal Care and Use Committee of Military Veterinary Institute (approval number: SCXK 2016-0008). All experiments with H5N6 viruses were performed in a biosecurity level 3 laboratory approved by the Military Veterinary Research Institute of the Academy of Military Medical Sciences.

### Viruses

Three H5N6 viruses were involved in this study; one was isolated from ducks, and two were isolated from chickens. The virus isolates were A/duck/Hubei/XY-01/2016 (abbreviated as DK01), A/chicken/Hubei/XY-165/2016 (abbreviated as CK165), and A/chicken/Hubei/XY-918/2016 (abbreviated as CK918). Viruses were grown in 9-day-old specific pathogen-free eggs (Merial Vital Laboratory Animal Technology Company, Beijing, China) and stored at −80 °C.

### Phylogenetic and mutation analyses

Viral RNA was extracted from allantoic fluid using TRIzol Reagent (Invitrogen Carlsbad, CA, USA) and reverse transcribed into cDNA using the primer Uni12 (5′-AGC RAA AGC AGG-3′). PCR products of H5N6 virus HA and NA fragments were amplified using specific viral primers as described by Hoffmann *et al*.^24^. The PCR products were purified and sequenced by Comate Bioscience Company Limited, and sequence data were analyzed with the SEQMAN program (DNASTAR, Madison, WI, USA). All reference sequences used in this study were obtained from the National Center for Biotechnology Information (NCBI) GenBank database. Phylogenic analysis was performed by the distance-based neighbor joining method using MEGA7.0.21 software (DNAStar, Inc.).

### Receptor-binding specificity assay

The receptor-binding specificities of the three H5N6 viruses were determined by HA assays with 1% cRBC and sRBC suspensions. For sialidase treatment, 90 μL of a 10% cRBC suspension was treated with 10 μL of α-2,3-sialidase (50 mU/μL) (TaKaRa, Dalian, China) for 10 min at 37 °C. The sample was then washed two times with PBS, centrifuged at 1500 rpm for 5 min each time, adjusted to a final working concentration (1%) with PBS, and stored at 4 °C. For VCNA (Roche, San Francisco, CA) treatment, 90 μL of a 10% cRBC suspension was treated with 10 μL of VCNA (50 mU/μL) for 1 h at 37 °C, washed two times with PBS, centrifuged at 1500 rpm for 5 min each time, adjusted to a final working concentration (1%) with PBS, and stored at 4 °C. For the HA assay, viruses were serially diluted 2-fold with 50 μL of PBS and mixed with 50 μL of a 1% RBC suspension in a 96-well plate. HA titers were read after 20 minutes of the reaction at room temperature.

### Mouse experiments

Groups of five six-week-old female BALB/c mice (Merial Vital Laboratory Animal Technology Company) were anesthetized with ether and intranasally inoculated with 50 μL of the H5N6 viruses at 10^6^ EID_50_. The weight loss and survival rates of mice in these groups were monitored daily for 14 days. The percentages of body weight change for each group were calculated by comparing the group average weight with their initial average weight. Mice that lost >25% of their original body weight were humanely euthanized.

To assess viral growth and pathological changes in the lungs of the infected mice, 12 mice per group were anesthetized with ether and intranasally inoculated with the H5N6 viruses at 10^6^ EID_50_, while another three mice intranasally inoculated with PBS served as the control. Three mice were euthanized at 1, 3, 5, and 7 days post-infection (dpi). The lungs of these mice were removed to determine the viral titers. Briefly, the lung tissues were weighed, and 0.1 gram of each tissue was placed into 1 ml of PBS containing 100 U/ml penicillin, making 10% weight/volume lung homogenates. The tissue samples were homogenized by Tissue Lyser (QIAGEN, Germany) and centrifuged at 12000 rpm. The supernatants were then collected and inoculated into 9-day-old embryonated eggs. After 72 h of incubation at 37 °C, the hemagglutinin activity was tested, and the EID50 was determined by the Reed method. At 5 dpi, the lungs of the infected mice were fixed in formalin, embedded in paraffin and stained with hematoxylin and eosin (H & E) for pathological examination.

### Guinea pig experiments

Hartley strain female guinea pigs weighing 300 to 350 g (Merial Vital Laboratory Animal Technology Company) were used in this study. In the transmission studies, three guinea pigs per group were intranasally inoculated with 200 μL of the test viruses at 10^6^ EID_50_ and housed in a cage placed inside an isolator. The next day, three naive guinea pigs were co-housed in the same cage with the three infected guinea pigs for the direct contact transmission studies, and another three naive guinea pigs per group were housed in a wire-frame cage adjacent to the infected guinea pigs for the aerosol transmission studies. The distance between the infected and aerosol-contact guinea pig cages was 5-cm. To monitor virus shedding, nasal washes were collected and titrated from all the animals at 2, 4, 6, and 8 dpi.

### Statistics analysis

Statistically significant differences were determined using one-way analysis of variance (ANOVA) with GraphPad Prism software (San Diego, CA, USA). All of the assays were run in triplicate and are representative of at least 3 separate experiments. The error bars represent the standard deviation.
