# Author Correction: Avian Influenza H5N6 Viruses Exhibit Differing Pathogenicities and Transmissibilities in Mammals

**DOI:** 10.1038/s41598-018-26658-0

**Published:** 2018-06-08

**Authors:** Zongzheng Zhao, Zhendong Guo, Chunmao Zhang, Lina Liu, Ligong Chen, Cheng Zhang, Zhongyi Wang, Yingying Fu, Jiaming Li, Huabin Shao, Qingping Luo, Jun Qian, Linna Liu

**Affiliations:** 1 Institute of Military Veterinary, Academy of Military Medical Sciences, 666 West Liuying Road, Changchun, 130122 Jilin, China; 2 College of Veterinary Medicine, Hebei Agricultural University, 2596 lucky south street, Baoding, 071000 Hebei, China; 3 Key Laboratory of Prevention and Control Agents for Animal Bacteriosis, Institute of Animal Husbandry and Veterinary, Hubei Academy of Agricultural Sciences, Wuhan, China

Correction to: *Scientific Reports* 10.1038/s41598-017-16139-1, published online 24 November 2017

The original version of this Article contained an error in the name of the author Jiaming Li, which was incorrectly given as Jianming Li. This has now been corrected in the PDF and HTML versions of the Article.

